# Bioinspired Super Thermal Insulating, Strong and Low Carbon Cement Aerogel for Building Envelope

**DOI:** 10.1002/advs.202300340

**Published:** 2023-04-24

**Authors:** Fengyin Du, Wenkai Zhu, Ruizhe Yang, Yun Zhang, Jiawei Wang, Weihuan Li, Wenqiang Zuo, Lizhi Zhang, Liuyan Chen, Wei She, Tian Li

**Affiliations:** ^1^ Jiangsu Key Laboratory of Construction Materials School of Materials Science and Engineering Southeast University Nanjing 211189 P. R. China; ^2^ School of Mechanical Engineering Purdue University West Lafayette IN 47906 USA; ^3^ State Key Laboratory of High‐Performance Civil Engineering Materials Jiangsu Sobute New Materials Co., Ltd. 211103 Nanjing P. R. China; ^4^ Center for High Performance Buildings Purdue University West Lafayette IN 47906 USA; ^5^ Department of Mechanical and Aerospace Engineering University at Buffalo The State University of New York Buffalo NY 14226 USA

**Keywords:** bioinspired materials, building envelop, material efficiency, mechanical strength, thermal insulation

## Abstract

The energy crisis has arisen as the most pressing concern and top priority for policymakers, with buildings accounting for over 40% of global energy consumption. Currently, single‐function envelopes cannot satisfy energy efficiency for next‐generation buildings. Designing buildings with high mechanical robustness, thermal insulation properties, and more functionalities has attracted worldwide attention. Further optimization based on bioinspired design and material efficiency improvement has been adopted as effective approaches to achieve satisfactory performance. Herein, inspired by the strong and porous cuttlefish bone, a cement aerogel through self‐assembly of calcium aluminum silicate hydrate nanoparticles (C‐A‐S‐H, a major component in cement) in a polymeric solution as a building envelop is developed. The as‐synthesized cement aerogel demonstrates ultrahigh mechanical performance in terms of stiffness (315.65 MPa) and toughness (14.68 MJ m^−3^). Specifically, the highly porous microstructure with multiscale pores inside the cement aerogel greatly inhibits heat transfer, therefore achieving ultralow thermal conductivity (0.025 W m^−1^ K^−1^). Additionally, the inorganic C‐A‐S‐H nanoparticles in cement aerogel form a barrier against fire for good fire retardancy (limit oxygen index, LOI ≈ 46.26%, UL94‐V0). The versatile cement aerogel featuring high mechanical robustness, remarkable thermal insulation, light weight, and fire retardancy is a promising candidate for practical building applications.

## Graphical abstract

Inspired by the strong and porous cuttlefish bone, a robust, durable, and thermal insulating aerogel through scalable self‐assembly of cement hydration nanoparticles (C‐A‐S‐H) is demonstrated. This bioinspired structural optimization addresses the longstanding dilemma in achieving both high mechanical strength and high porosity for good thermal insulation.

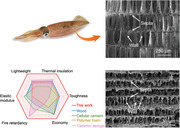

## 1 Introduction

The energy crisis is considered a critical issue by policymakers and is given high priority due to its potential to affect various aspects of society.^[^ ^1^, ^2^ ^]^ Building sector around the world takes up over 40% of the global energy consumption and is one of the largest producers of greenhouse gas.^[^ ^3^, ^4^ ^]^ Such energy consumption of buildings is mainly due to the heating and cooling systems required for thermal comfort within the building envelope.^[^ ^5^, ^6^ ^]^ Heat conduction, as one of the primary heat transfer pathways, through walls and roofs accounts for the largest component of a building's total thermal load, i.e., 40% of total building energy system in a typical household.^[^ ^7^ ^]^ Currently, commercial insulation materials including mineral wool, glass fiber, and polystyrene foam, have a thermal conductivity within 0.030–0.090 W m^−1^ K^−1^.^[^ ^8^, ^9^ ^]^ Such performance of these conventional insulating materials cannot satisfy energy efficiency for the next‐generation buildings. Advanced thermal insulators like aerogels are a class of porous and lightweight materials with ultralow thermal conductivity.^[^ ^10^ ^]^ However, these highly porous materials (including organic and inorganic aerogel) with good thermal insulation often have a compromised mechanical strength and additional structural materials like cement and concrete are needed for reinforcement.^[^ ^11^ ^]^

To integrate structural support, thermal insulation, and more functionalities in a single building material, bioinspiration and biomimicking are adopted to further optimize building materials as a promising route. For example, by mimicking the cellular structure of honeycombs, lightweight foamed concretes are able to confine heat flow through tortuous pores and have been successfully applied as building facades.^[^ ^12^ ^]^ Inspired by the natural nacre architecture, cement composites with “brick‐mud” structures significantly improved mechanical strength and toughness at the same time without aggravation in density.^[^ ^2^, ^13^ ^]^ However, mass‐produced building materials are required to minimize carbon footprints in the era of carbon control. Therefore, how to improve material efficiency to gain higher performance with less raw material is the critical challenge for the next generation of building materials.

**Figure 1:**
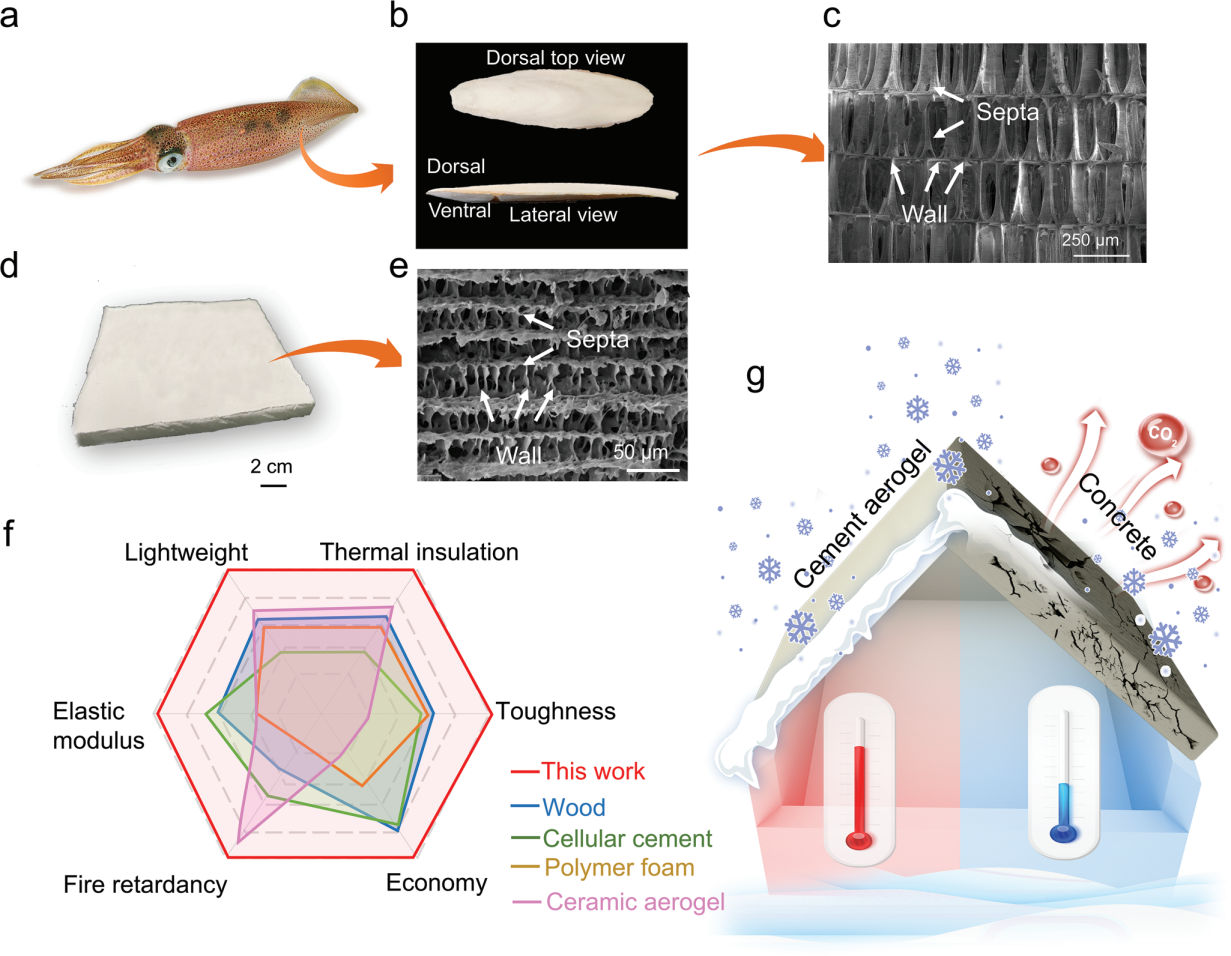
Bioinspired cement aerogel. a) Photo of cuttlefish. b) Top and lateral view of cuttlefish bone. c) SEM showing the microstructure of cuttlefish bone. d) Large‐scale cement aerogel of 10 cm × 10 cm. e) SEM showing the microscopic morphology of cement aerogel. f) Performance comparison of cement aerogel, wood, cellular cement, polymer foam and ceramic aerogel as building materials.^[^ ^19^, ^20^, ^21^, ^22^ ^]^ g) A schematic comparison of the energy saving process while using the cement aerogel (left half) and concrete (right half) as building envelope: cement aerogel is lighter, more thermally insulating and more fire resistant than concrete.

Cement as the most widely used building material in the world is a promising candidate for such multifunctional innovation because of its high mechanical strength, long‐term durability, and high‐temperature resistance. As a major component in cement, calcium aluminum silicate hydration (C‐A‐S‐H) is largely abundant and provides ultrahigh cohesive force to drive cement into solid binding blocks.^[^ ^14^, ^15^ ^]^ To improve material efficiency, C‐A‐S‐H nanoparticles are self‐assembled in polyvinyl alcohol (PVA) solution (referred to as cement aerogel), which avoids the calcination of cement raw materials and leads to over 50% reduction in carbon emissions compared to cement.^[^ ^16^ ^]^ To comply with the goals of simultaneous structural robustness and thermal insulation, we bio‐mimic the strong and porous bone structure of cuttlefish and develop this versatile building material. The cuttlefish bone with a ≈93% porosity can withstand external water pressure as high as 20 atm. in the deep sea. The lightweight cuttlebone can realize high impact absorption and good thermal insulation through “wall‐septa” microstructure (**Figure** **1**a–c).^[^ ^17^, ^18^ ^]^ In the cement aerogel design, C‐A‐S‐H improves the cross‐link of polymer chains and generates an effective protective layer on the burning surface, which simultaneously enhances the mechanical strength and fire retardancy. The proposed developed synthesis method allows a scalable fabrication of the cement aerogel (10 cm × 10 cm) with microstructure resembling the cuttlefish bone (Figure 1d,e). The cement aerogel displays unique and comprehensive properties of low density, high mechanical stiffness (315.65 MPa), high toughness (14.68 MJ m^−3^), low thermal conductivity (0.025 W m^−1^ K^−1^)), and fire retardancy (LOI: 46.26%, UL94‐V0), indicating the advances of coupled resistance compared to conventional building materials (Figure 1f). In addition, the highly porous cement aerogel greatly improves the material efficiency, i.e., using less materials with higher performance. The large‐scale deployment can also mitigate the greenhouse gas emission associated with conventional cement industry. The bioinspired design address the long‐term dilemma achieving high mechanical strength and high thermal insulation at the same time, offers new ventures to engineer novel building materials and address the inherent coupling problems of mechanical and thermal properties in advanced building envelops (Figure 1g).

## 2 Results and Discussion

**Figure 2:**
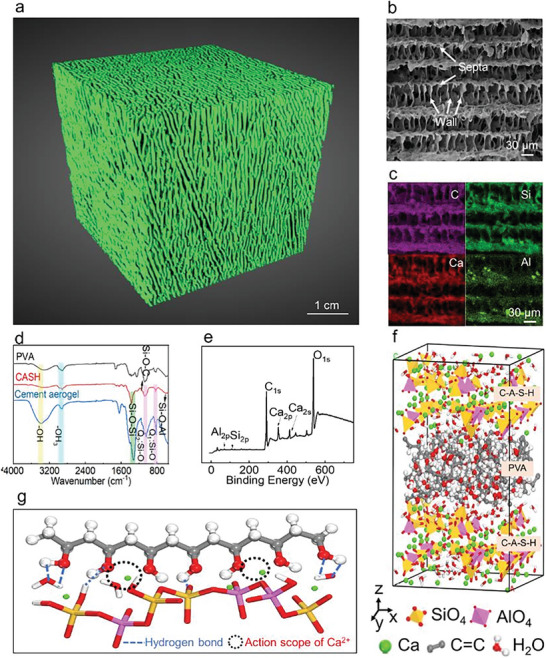
Design and microstructure of the cement aerogel. a) XCT of the cement aerogel. b) SEM image of the cement aerogel with walls and septa. c) EDS maps of cement aerogel showing the detected elements (carbon, silicate, calcium, and aluminum). d) FTIR spectrum of PVA, CASH and cement aerogel. e) XPS of cement aerogel. f) Panorama of simulated cement aerogel. g) Magnified schematic illustration of the interface between PVA chains and C‐A‐S‐H particles.

The scanning electron microscope (SEM) captures the sophisticated architecture of cuttlefish bone in Figure 1c. The cuttlefish bone consists of the lamellar “wall‐septa” structure, with around 100 µm in thickness, 80 µm in spacing. Imitating the aligned structure of cuttlefish bone, cement aerogel is fabricated in two steps, where C‐A‐S‐H nanoparticles are firstly self‐assembled in PVA solution and then the well‐dispersed suspension is directionally freeze dried (Figure S1, Supporting Information) (see Experimental Section). X‐ray computed microtomography (XCT) images of bioinspired cement aerogel show the macroscopically well‐aligned lamellar porous structure in **Figure** **2**a and the 3D rotation in Movie S1 (Supporting Information). The cuttlefish bone‐like structure is further illustrated by the SEM images (Figure 2b). The microstructure of cement aerogel consists of 20–40 µm interlayer spacing and 2–4 µm layer thickness, corresponding with a porosity of 90.43% and the minimum density of 0.015 g cm^−3^ (Table S1, Supporting Information). The relevant energy dispersive spectrum (EDS) mapping results verify the key element composition (Figure 2c). The mappings of Ca, Al and Si elements almost overlap with that of C elements, suggesting the C‐A‐S‐H nanoparticles are evenly distributed on the surface of PVA skeleton. In this way, the C‐A‐S‐H nanoparticles effectively form a protective layer upon polymeric skeleton surface.

The self‐assembly C‐A‐S‐H nanoparticles along PVA chains have pore diameter of 20 nm and PVA chains are cross‐linked by divalent metal ion from C‐A‐S‐H gel (Figure S2, Supporting Information). Upon cross‐bonding with PVA in water, the C‐A‐S‐H gel and PVA chains form strong networks and are well dispersed because of mutual ion exchange (Figure S3, Supporting Information). The formation of C‐A‐S‐H nanocrystals can be detected by x‐ray diffraction (XRD) analysis with the typical characteristic peak at 29.1° (Figure S4, Supporting Information). The comparison of Fourier transforms infrared spectrometer (FTIR) among C‐A‐S‐H, PVA and cement aerogel is shown in Figure 2d. The strong Si—O—Si asymmetric stretching vibrations at 1330 cm^−1^ and 917 cm^−1^, as well as a relatively weak peak of Si—O—C bond at 1150 cm^−1^ are detected, demonstrating the chemical interactions between the PVA and the C‐A‐S‐H. A new and wide peak can be found at 617 cm^−1^ in the spectrum of the mixture, which can be ascribed to the formation of new Si—O—Al bond in C‐A‐S‐H. X‐ray photoelectron spectroscopy (XPS) results also demonstrate the coordination interactions of calcium, silicate, and aluminum with PVA chains (Figure 2e and Figure S5, Supporting Information), as calcium exists as Ca^2+^ (350.28 eV) and Ca—O (346.48 eV), silicon exists as Si—O (99.38 eV) and aluminum exists as Al—O (72.38 eV) (Figure S5, Supporting Information). The molecular dynamics (MD) simulation is applied to investigate the molecular interactions between PVA chains and C‐A‐S‐H nanoparticles (Figure 2f,g). The molecular structures of cement aerogel are built and stretched along the z‐axis. The calcium ions in the interlayer not only coordinate with the oxygen atoms from silicates but also attract oxygen from PVA groups (Figure 2g, dashed black circles).^[^ ^14^, ^23^ ^]^ Figure S6 (Supporting Information) also proves these strong interfacial forces where the major peaks located around 2.25 Å can represent the strong hydrogen correlation of PVA and C‐A‐S‐H, and the peak at 3.1 Å represents the spatial interaction between Ca^2+^ and O_nb_. Therefore, the interface of PVA chains and C‐A‐S‐H nanoparticles are tightly bonded, which results in many superior properties.

### 2.1 Mechanical Performance and Negative Poisson's Ratio

**Figure 3:**
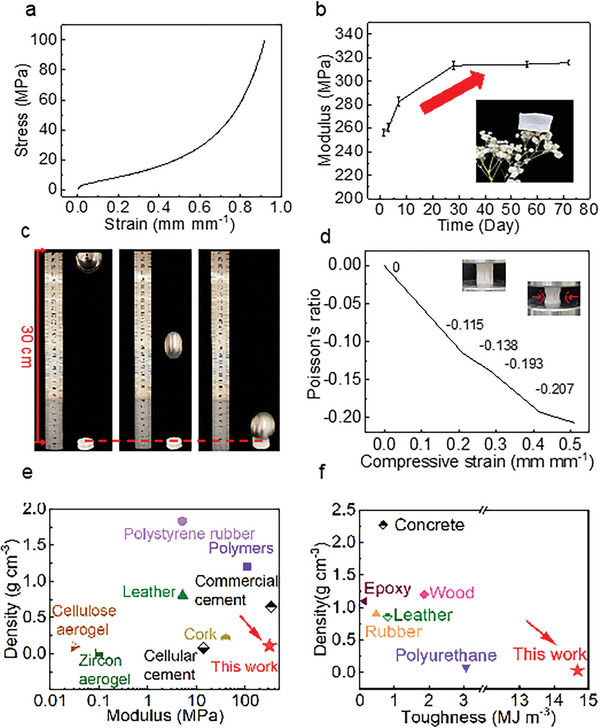
Mechanical performance of cement aerogel. a) Stress–strain curve under axial compression test of cement aerogel. b) Aging test of cement aerogel, indicating the increase in mechanical strength, inset image of the lightweight cement aerogel with 0.015 g cm^−3^ in Figure S7 (Supporting Information). c) A set of real‐time images of impact load on cement aerogel. d) Poisson's ratio of cement aerogel as a function of compressive strain. e) Comparison of density and modulus among cement aerogel and other commercial materials.^[^ ^22^, ^24^, ^25^, ^26^, ^27^, ^28^, ^29^, ^30^ ^]^ f) Toughness versus density for the cement aerogel and other commercial materials.^[^ ^20^, ^25^, ^31^, ^33^ ^]^

To protect buildings from mechanical crushing, high stiffness and strong compression and impact resistance are the most desirable advantages. Due to the solid skeleton and high strength of cement, the acquired cement aerogel can tolerate large compression without cracking and deformation. The measured stress‐strain curve under axial compression test in **Figure** **3**a shows the typical foam‐like deformation behavior. The axial compressive curve followed the three characteristics of open honeycomb‐like foams: an elastic regime, a stress plateau, and high densification, acquiring a large elastic modulus of 255.98 ± 2.89 MPa. In the aging test, the mechanical stiffness increased with the increasing time (from 255.98 ± 2.89 MPa to 315.65 ± 2.17 MPa, increasing 23.52% in the following 72 d) in Figure 3b. The compression strength has a similar trend (from 59.46 ± 3.50 MPa to 73.32 ± 2.04 MPa at 80% strain, increasing 23.31% in Figure S8, Supporting Information). The increase of the mechanical strength of cement aerogel over a long period is due to the 1) hardening and chemical stability of C‐A‐S‐H particles^[^ ^33^, ^34^, ^35^, ^36^ ^]^ and 2) strong interface bonding between C‐A‐S‐H and PVA skeleton (Figure S9, Supporting Information). The contact angle of the as‐processed cement aerogel is 48.9° suggesting hydrophilicity (Figure S10a,b, Supporting Information). Figure S10c (Supporting Information) shows an increase in its moisture absorption under 100% RH (20.6 °C) due to the hydrophilic nature of PVA. To address the wettability concern, one possible method is applying a nanosilica (SiO_2_) coating to increase the hydrophobicity of cement aerogel. After the coating, the contact angle of cement aerogel increased to 109.7 °, indicating a hydrophobic surface shown in Figure S10d,e (Supporting Information). And the weight change of cement aerogel in Figure S10f (Supporting Information) indicates negligible moisture absorption.

Benefiting from the rigid framework, cement aerogel demonstrates ultrahigh toughness through drop‐tower impact test. A solid steel ball (200 g, 200 times heavier than the cement aerogel sample) was repeated to release from a height of 300 mm (19 times higher than the sample) to show the anti‐impact protection of cement aerogel (Figure 3c,d, Movie S2, Supporting Information). After the third release, cement aerogel still maintained its structural integrity without any visible cracks. The microstructure of cement aerogel before and after repeated impact loads are provided in Figure S11 (Supporting Information). The bionic “septa‐wall” microstructure remains almost the same after impact loads, potentially due to the enhanced skeleton by C‐A‐S‐H and the strong interface between C‐A‐S‐H and PVA. The mechanical stiffness of this sample after impact loads is 294.61 MPa (Figure S12, Supporting Information). The toughness of cement aerogel is calculated by integrating as high as 14.68 MJ m^−3^ at 40% strain. Moreover, the negative transverse strains under compressive load are indicated by the negative Poisson's ratio during axial compression. Figure S13 and Movie S3 (Supporting Information) recorded the real longitudinal and transverse strain evolution of cement aerogel under loading. The longitudinal strain decreases from 0 to 0.51, accompanied by the transverse strain decreasing from 0 to ‐0.11, and thus Negative Poisson's ratio (NPR) was calculated at ‐0.22 in Figure 3e. The schematic and microscopic diagrams in Figure S14 (Supporting Information) illustrate the mechanical anisotropy and the failure modes of cement aerogel. At the axial direction, the septa frame collapsed inward and led to a macroscopic Negative Poisson's ratio of cement aerogel. At the radial direction, the “wall” skeleton in multiple layers serves as a direct support to bear the external load. The layer‐by‐layer failure of the septa frame leads to periodic fluctuations in Figure S14d (Supporting Information). The growth patterns of directional ice crystals are depicted in Figure S15 (Supporting Information), along with the corresponding mechanical property. Cement aerogel has a much higher specific modulus compared to other low‐density materials (<2.0 g cm^−3^), the cement aerogel with a specific modulus of 2895.87 MPa cm^3^ g^−1^ is three orders of magnitude higher than that of cellulose aerogel with 3.33 MPa cm^3^ g^−1^ and an order of magnitude higher than that of commercial cement with 524.62 MPa cm^3^ g^−1^ (Figure 3f).^[^ ^27^, ^30^ ^]^ It exhibits the highest toughness (14.68 MJ m^−3^), one order of magnitude higher than that of wood with 3.7 MJ m^−3^.^[^ ^20^ ^]^ The creeping results of cement aerogel are shown in Figure S16 (Supporting Information). Under a long‐term constant compression, the strain increased at the beginning and finally stabilized within 8%. This creeping behavior is generated by the viscous flow of C‐A‐S‐H particles inside cement aerogel, which can relieve the internal stress and effectively prevent cracking.

Furthermore, unlike the production of traditional cement, which emits 3 tons of CO_2_ per cubic meter, the method we developed only releases about 160 kg of CO_2_ as it avoids the calcination of cement raw materials, which means a significant reduction of 94.7% in carbon emissions.^[^ ^37^ ^]^

### 2.2 Thermal Insulation and Fire Retardancy Property

**Figure 4:**
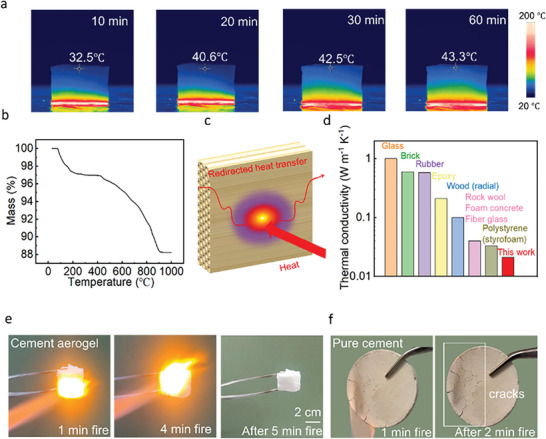
Thermal and fire retardancy of cement aerogel. a) Infrared images of the cement aerogel during hot plate experiment. b) The thermal gravimetric (TGA) analysis of cement aerogel. c) Schematics of thermal insulation performance of cement aerogel. d) Thermal conductivity comparison of cement aerogel and commercial materials.^[^ ^5^, ^25^, ^38^, ^39^ ^]^ e) Photos of cement aerogel under fire. f) Photos of pure cement under fire.

Good thermal insulation is desirable for structural materials to reduce energy consumption in residential and commercial buildings. Highly porous architecture possesses better thermal insulation than solid structures because the pore volume and multiple internal gaps reduce heat conduction mediums. Thermal insulation performance of cement aerogel is demonstrated by a hot plate method. As shown in **Figure** **4**a, a gradient distribution of temperature through cement aerogel was observed when heating on the 200 °C hot plate. The surface temperature of cement aerogel (12 mm) was relatively low at 40.6 °C after 20 min heating and increased slightly to 42.5 °C after 30 min and remained constant thereafter. While the surface temperature of pure cement sample increased to 200 °C dramatically after 10 min heating, which exhibits poor thermal insulation property without bioinspired structure (Figure S17, Supporting Information). The thermal gravimetric (TGA) analysis shows that cement aerogel exhibits great thermal stability and remains 88.25% mass after 1000 °C heating, which suggests cement aerogel can maintain structural integrity at high temperature of 1000 °C. The ultralow thermal conductivity of cement aerogel is attributed to the multiscale pore size (ranging from 20 nm to 50 µm) and large porosity of cement aerogel (Figure S18, Supporting Information). When thermal energy is transported onto the cement aerogel, the hierarchal structure of aligned skeleton effectively redirects the absorbed heat in the planar direction (Figure 4c).^[^ ^29^ ^]^ Furthermore, it is noteworthy that our cement aerogel gained both high thermal insulation (accompanied with high porosity) and high mechanical strength at the same time, which are mutually exclusive in traditional ceramic building materials. Compared to commercial insulation building materials (with thermal conductivity of 0.05 W m^−1^ K^−1^), the cement aerogel exhibited superior insulation with the low thermal conductivity of 0.025 W m^−1^ K^−1^) in Figure 4d and Table S2 (Supporting Information). Such coordination between heat insulation and impact absorption in cement aerogel is benefited from the well‐designed bionic structure and strong bonding interface among organic–inorganic composite.

Modern building facilities must face the threat of fire disasters, which have caused significant economic loss to society. To verify reliable fire protection, several flammability tests were carried out on cement aerogel including LOI experiment, vertical burning test (UL94) and burning tests. The LOI experiment was also used to assess the fire retardancy properties of the cement aerogel. The cement aerogel has a significantly higher LOI value (46.26%) than commercial foams (22%).^[^ ^40^, ^41^ ^]^ To demonstrate the persistent resistance of fire, the cement aerogel was burning under butane blowtorch for over 5 min and the structure remained sturdy (Figure 4e and Movie S4, Supporting Information). On the contrary, there are many obvious cracks on cement surface after 1 min fire, which results from the volume expands from uncomplicated hydrated cement (Figure 4f). In infrared images in Figure S19 (Supporting Information), the top surface of cement aerogel was able to keep at a low temperature at 33.6 °C after 5 min fire exposure. The great flame retardancy of the cement aerogel can be attributed to the inorganic C‐A‐S‐H nanoparticles generating an effective protective layer on the burning surface of the laminar walls. The high flame retardancy of cement aerogel can be attributed to the presence of inorganic C‐A‐S‐H nanoparticles, which generate a highly effective protective layer on the burning surface of the laminar walls. This is due to the ceramic composition of C‐A‐S‐H particles, which are naturally fire‐resistant owing to their temperature stability and low thermal conductivity. Moreover, the optimal mass ratio of organic PVA to inorganic C‐A‐S‐H in cement aerogel is approximately 2:3, as this ratio has been demonstrated to enhance both the mechanical properties and flame retardancy of the material. The good flame retardancy of the cement aerogel can significantly delay the spread of the fire and provides enough time to for escaping and rescuing in extreme environments.

## 3 Conclusion

In summary, we have developed a bioinspired cement aerogel with great improvement in material efficiency through self‐assembling the major component of cement (C‐A‐S‐H nanoparticles) in polymer solution. Our process method avoids the calcination of cement raw material, leading to a reduction in embodied carbon by over 50% compared with regular cement. The resulting cement aerogel exhibits a mass density of only 0.015 g cm^−3^. The bioinspired structural optimization addresses the dilemma in high mechanical strength (stiffness up to 315.65 MPa, toughness 14.68 MJ m^−3^) and high porosity. The multiscale pore sizes (from 20 nm to 50 µm) and large porosity help to obtain the low thermal conductivity (as low as 0.025 W m^−1^ K^−1^). In addition, the inorganic C‐A‐S‐H nanoparticles in cement aerogel form a protective barrier against fire for a high fire retardancy (LOI: 46.26%, UL94‐V0). The ultralight and highly porous cement aerogel featuring high mechanical strength, remarkable toughness, thermal insulation, and fire retardancy is a promising candidate for the next generation of building materials. This super thermally insulating and mechanically strong cement aerogel has several advantages as an energy‐efficient material for vehicles and space, offering a reduced environmental impact and cost‐effectiveness compared to traditional aerogel. Its high performance makes it a competitive alternative to cement in buildings and expands its application to structural construction.

## 4 Experimental Section

### Materials

Polyvinyl alcohol (PVA ≈ 224, Mw ≈ 205 000), calcium nitrate (Ca(NO_3_)_2_·4H_2_O), aluminum nitrate (Al(NO_3_)_3_·9H_2_O), and sodium silicate (Na_2_SiO_3_·9H_2_O) were provided by Shanghai Macklin Biochemical Co., Ltd.

### Fabrication of the Cement Aerogel

The spinning solution was prepared from four precursors: PVA, Ca(NO_3_)_2_·4H_2_O, Na_2_SiO_3_·9H_2_O and Al(NO_3_)_3_·9H_2_O. To begin the typical synthesis, 10 g PVA was dissolved in 100 g deionized water by magnetic stirring at 90 °C for 1 h. Then, 50 g Ca(NO_3_)_2_ solution (1 m) was added to the PVA solution for cross‐linking. Next, 40 g Na_2_SiO_3_ (1 m) and 10 g Al(NO_3_)_3_ (1 m) solutions were sequentially added to generate in‐situ C‐A‐S‐H nanoparticles. This composition was used consistently for all the characterizations, including mechanical strength, thermal insulation and fire retardancy.

The chemical equation can be expressed as:

(1) $${\mathrm{Ca}}^{2+}{+\mathrm{SiO}}_{3}^{2-}+Al^{3+}+H_{2}O\to {\left(\mathrm{Cao}\right)}_{x}\left({\mathrm{Al}}_{2}O_{3}\right){\mathrm{SiO}}_{2}{\left(H_{2}O\right)}_{y}$$

which results in the formation of C‐A‐S‐H nanoparticles.

The transparent solution gradually became milky white with the addition of sodium silicate, which means the generation of C‐A‐S‐H nanoparticles. All procedures were performed at ambient temperature. After C‐A‐S‐H nanoparticles were synthesized in PVA liquid solution (Figure S1a, Supporting Information), the fresh mixture was frozen in liquid nitrogen bath, where PVA gel with C‐A‐S‐H nanoparticles was expulsed and assembled by the controlled phase separation as ice crystalizes from the bottom (Figure S1b, Supporting Information). After coalescence and solidification (Figure S1c, Supporting Information), the templated ice was sublimated in freezing drying at ‐110 °C and 1 Pa for 48 h to preserve the cuttlefish‐like microstructure of cement aerogel (Figure S1d, Supporting Information).

### Characterizations

The compressive tests were performed using a universal UTM5105 testing machine with a 5000 kN load cell. The loading rate was set at 3 mm min^−1^. The microscopic morphologies of the aerogel were examined with a field emission scanning electron microscope (ZEISS Gemini 300, Germany). The elemental composition was examined with energy‐dispersive spectroscopy. The distribution of layered microstructures was calculated by analyzing the SEM images using Image‐Pro Plus software (Media Cybernetics, USA). The thermal conductivity of cement aerogel was measured by thermal conductivity analyses (TPS 2500S, Hot Disk Instruments, Sewden) with the transient hot plate method according to the testing standard of ISO 22007‐2: 2015. The samples were crushed into small fragments for an X‐ray diffractometer (Burker D8 Advance X, Germany) equipped with Cu K*α* radiation (*λ* = 0.15406 nm) with 2*θ* ranging from 5° to 80° at a 10° min^−1^ scanning rate. The density of the cement aerogel was calculated using the measured mass and geometry of each sample. Infrared (IR) spectra were recorded with an FTIR spectrometer (Bruker Vertex 70) at room temperature. The viscosity rate was determined by a rheometer (Physica MCR 302, Anton Paar, Austria) with a 0.5 N prestress and 1% shear strain at an angular frequency of 0.5–200 rad s^−1^. The parameters related to porosity and tortuosity were measured by a Mercury Porosimeter (Auto pore IV 9520, USA). The infrared thermal images of the samples were captured by an infrared (IR) thermal camera (FLTR T540).

### Molecular Dynamics (MD) Simulations

The model cube was set up with dimensions of *a* = 21.38 Å, *b* = 20.60 Å, and *c* = 34.26 Å. A large‐scale atomic/molecular massively parallel simulator (LAMMPS) was utilized to carry out the simulation. In this simulation, two force fields, the clay force field (CLAYFF) and the consistent valence force field (CVFF), were packed into the interactions during the simulation process. Specifically, the former is suitable for the analysis of C‐S‐H, and the latter is used to describe PVA. The interaction parameters between C‐S‐H and PVA atoms were calculated based on the mean rule, including the arithmetic mean rule for distance parameters and the geometric mean rule for energy parameters. Additionally, a hood canonical ensemble (NVT) with 298 K was chosen in the whole simulation. The production period lasted 500 ps after a 100 ps equilibration time, and the data were collected every 1 ps during this period to investigate the interactions.

### Simulation Of Physical Process

The finite element method (FEM) simulation was implemented in ABAQUS software. A porous structure with a 50 cm length × 50 cm height × 3 cm weight was created to carry out the NPR behavior under the deformation process in the solid mechanical field.

## Conflict of Interest

The authors declare no conflict of interest.

## Supporting information

Supporting Information

Supplemental Movie 1

Supplemental Movie 2

Supplemental Movie 3

Supplemental Movie 4

## Data Availability

The data that support the findings of this study are available in the Supporting Information of this article.
