# Supplementary material for: Bioinspired Super Thermal Insulating, Strong and Low Carbon Cement Aerogel for Building Envelope
Source: Adv Sci (Weinh). 2023 Apr 24;10(18):2300340. doi: 10.1002/advs.202300340 (PMC10288245; doi:10.1002/advs.202300340)
Supplement: Supplementary file 1 — Supporting Information [file ADVS-10-2300340-s001.pdf]

ADVANCED SCIENCE
Open Access

Supporting Information

for *Adv. Sci.*, DOI 10.1002/advs.202300340

Bioinspired Super Thermal Insulating, Strong and Low Carbon Cement Aerogel for Building Envelope

Fengyin Du, Wenkai Zhu, Ruizhe Yang, Yun Zhang, Jiawei Wang, Weihuan Li, Wenqiang Zuo, Lizhi Zhang, Liuyan Chen, Wei She and Tian Li**

Supplementary Information

Table of contents:

Part 1: Supplementary Figures

Figure S1. Schematic of directional freeze drying. a) The *in-situ* synthesized C-A-S-H nanoparticles in PVA solution. b) Expulsed monomer by controlled ice crystal crystallizations. c) Coalescence and solidification. d) Ice template sublimation without destroying the microstructure.

Figure S2. SEM picture of calcium aluminum silicate hydrate (C-A-S-H) nanoparticles. The C-A-S-H nanoparticles have a pore diameter of 20 nm.

Figure S3. The picture of cement aerogel samples. a) The Polyvinyl Alcohol (PVA) transparent solution. b) The Na_2SiO_3 solution in PVA solution. c) The self-assemble C-A-S-H nanoparticles in PVA solution.

Figure S4. XRD picture of cement aerogel. There is a typical peak at 29.1° showing the generation of C-A-S-H nanoparticles.

Figure S5. XPS results of cement aerogel for a all elements. b) Si_{2p}. c) Al_{2p}. d) Ca_{2p}.

Figure S6. MD simulation results of cement aerogel. a) The radial distribution functions from the model in this simulation: -ho(PVA): hydrogen atoms from PVA chains, -oh(C-S-H):oxygen atoms from C-S-H, -oh(PVA): oxygen atoms from PVA chains, -ho(C-S-H): hydrogen atoms from C-S-H, -o*: oxygen atoms from water, -h*: hydrogen atoms from water, -ca(C-S-H): calcium atoms from C-S-H. b) Snapshots of the connections between PVA and C-S-H, green dotted circle are Ca-O coordination.

Figure S7. Cement aerogel shows ultralight weight with a density of 0.015 g cm^{-3}.

Figure S8. The durability of cement aerogel. Cement aerogel has good long-term durability: compression strength increases from 59.46 MPa to 73.32 MPa.

Figure S9. SEM images of cement aerogel interface, showing nanoparticles are tightly bonded on the PVA surface.

Figure S10. Before coating: a) contact angle. b) micro-optical photo. c) weight change under 100% RH. After coating: d) contact angle. e) micro-optical photo. f) weight change under 100% RH.

Figure S11. SEM images of cement aerogel a) b) before impact loads, c) d) after impact loads, showing no obvious cracks after impact loads.

Figure S12. The strain and stress curve of cement aerogel, showing a mechanical stiffness of 294.61 MPa.

Figure S13. The real-time longitudinal strain evolution of cement aerogel. The sequential

pictures show Negative Poisson's ratio (NPR) of cement aerogel under compression test. NPR as high as -0.22 can be clearly observed from compression tests.

Figure S14. The anisotropic mechanical property of cement aerogel. At the radial direction: a) Schematic of mechanical failure. b) Negative Poisson's ratio behavior. c) Strain and stress curve under compression. At the axial direction: d) Schematic of mechanical failure. e) Sequential SEM images of in situ compressed cement aerogel. f) Strain and stress curve under compression.

Figure S15. a) Schematic of 3D controlled ice freezing template. b) Schematic of controlled ice crystals in y-z view. c) Radially prepared sample in x-y view. d) Strain-stress curve of cement aerogel under compression: liner elasticity, plateau and high densification. The elastic modulus of this sample is 305.80 MPa. The compressive strength at 80% strain is 58.86 MPa. The toughness of this cement aerogel is 3.5 MJ m^{-3} at 15% strain.

Figure S16. Creeping behavior of cement aerogel, the creep behavior is generated by the viscous flow of C-A-S-H particles, which can relieve the internal stress and effectively prevent cracking.

Figure S17. The infrared images of pure cement samples during hot plat experiment. a) 0 min after 200 °C heating. b) 10 min after 200 °C heating.

Figure S18. SEM pictures of cement aerogel. a) SEM picture of cement aerogel at micro scale. b) SEM picture of cement aerogel at nano scale. Cement aerogel has two levels pores: the skeleton with layer spacing of 20-50 μm and the C-A-S-H nanoparticles with 10-50 nm space wrapped on the surface of skeleton.

Figure S19. Infrared images of the cement aerogel under fire, showing a low temperature at 33.6 °C after 5-minute fire exposure.

Part 2: Tunability Section

Note S1 Experimental Section

Table S1 The composition of cement wood.

Table S2 The density and porosity of different cement aerogel.

Table S3 The performance results of different cement aerogel.

Part 3: Table

Table S4 The thermal conductivity of different commercial insulations.

Part 4: Supplementary Movies

Movie S1. The 3D reconstruction of cement aerogel through x-ray computed microtomography (XCT)

Movie S2. The compression test of cement aerogel, the clear NPR behavior can be observed.

Movie S3. The impact load test of cement aerogel. When a 200 g steel ball falls from 30 cm height three times, there is no visible crack can be seen in cement aerogel.

Movie S4. The burning test of cement aerogel. After 5 min fire, there is no apparent damage in cement aerogel.

Part 1: Figures

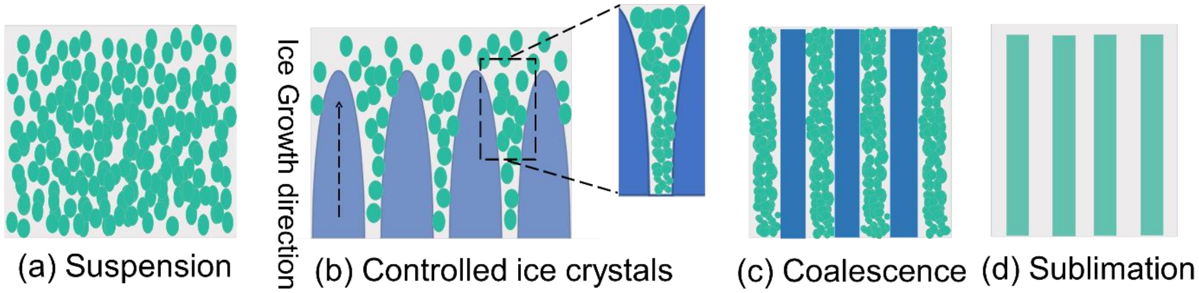

Figure S1. Schematic of directional freeze drying. a) The *in-situ* synthesized C-A-S-H nanoparticles in PVA solution. b) Expulsed monomer by controlled ice crystal crystallizations. c) Coalescence and solidification. d) Ice template sublimation without destroying the microstructure.

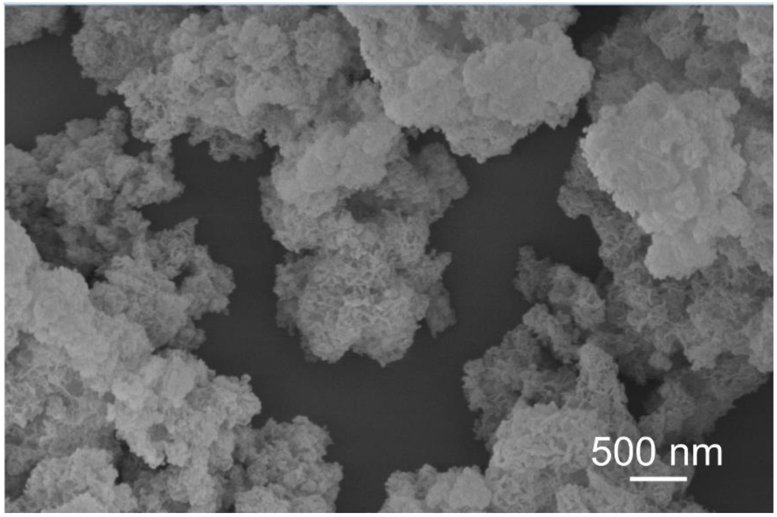

Figure S2. SEM picture of calcium aluminum silicate hydrate (C-A-S-H) nanoparticles. The C-A-S-H nanoparticles have a pore diameter of 20 nm.

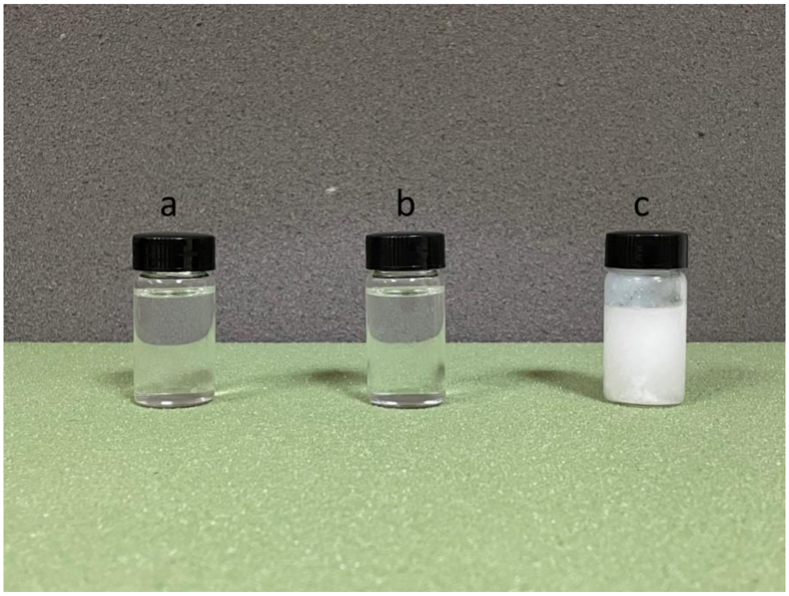

Figure S3. The picture of cement aerogel samples. a) The Polyvinyl Alcohol (PVA) transparent solution. b) The Na_2SiO_3 solution in PVA solution. c) The self-assemble C-A-S-H nanoparticles in PVA solution.

The resulting homogenized sol-gel dispersion (self-assemble C-A-S-H gel in PVA solution) could remain stable without gravity sedimentation. It shows a great combination of nanoparticles and PVA chains.

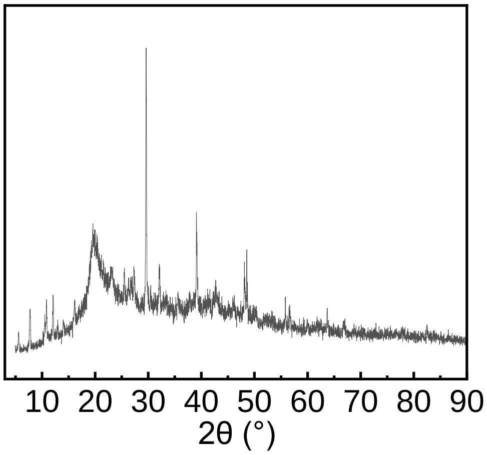

Figure S4. XRD picture of cement aerogel. There is a typical peak at 29.1° showing the generation of C-A-S-H nanoparticles.

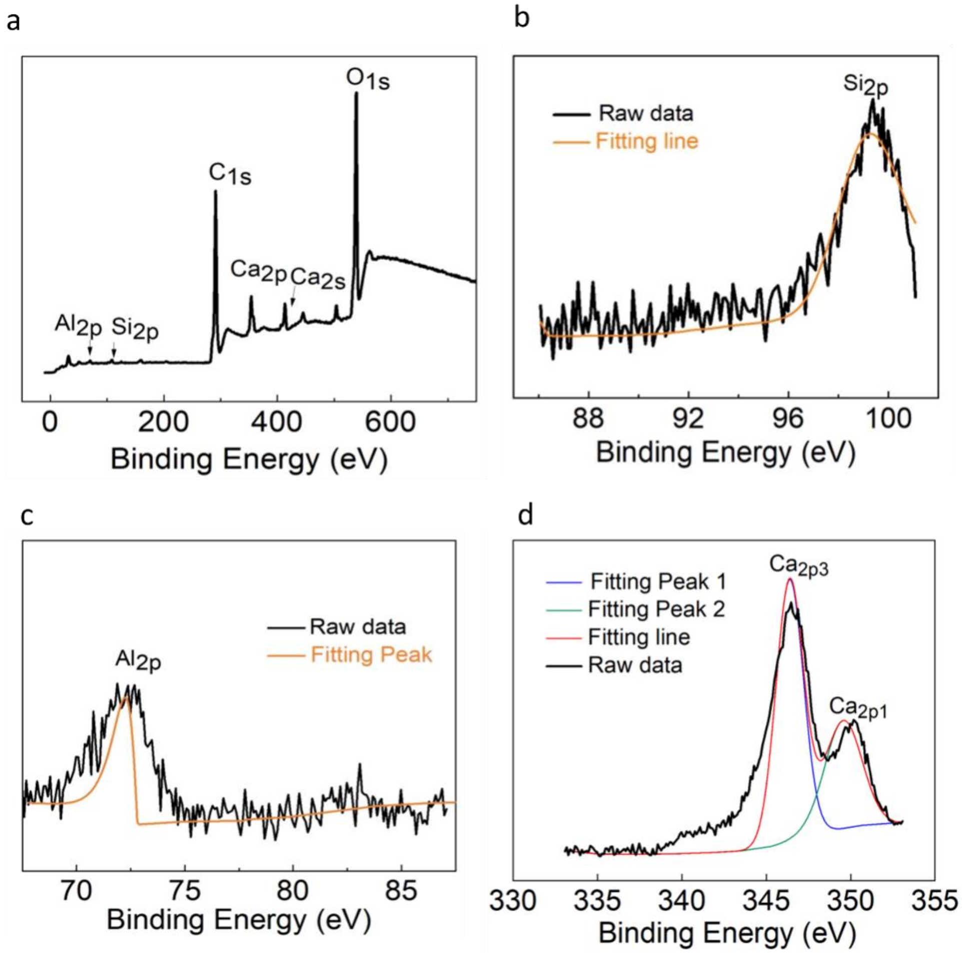

Figure S5. XPS results of cement aerogel for a) all elements. b) Si_{2p}. c) Al_{2p}. d) Ca_{2p}.

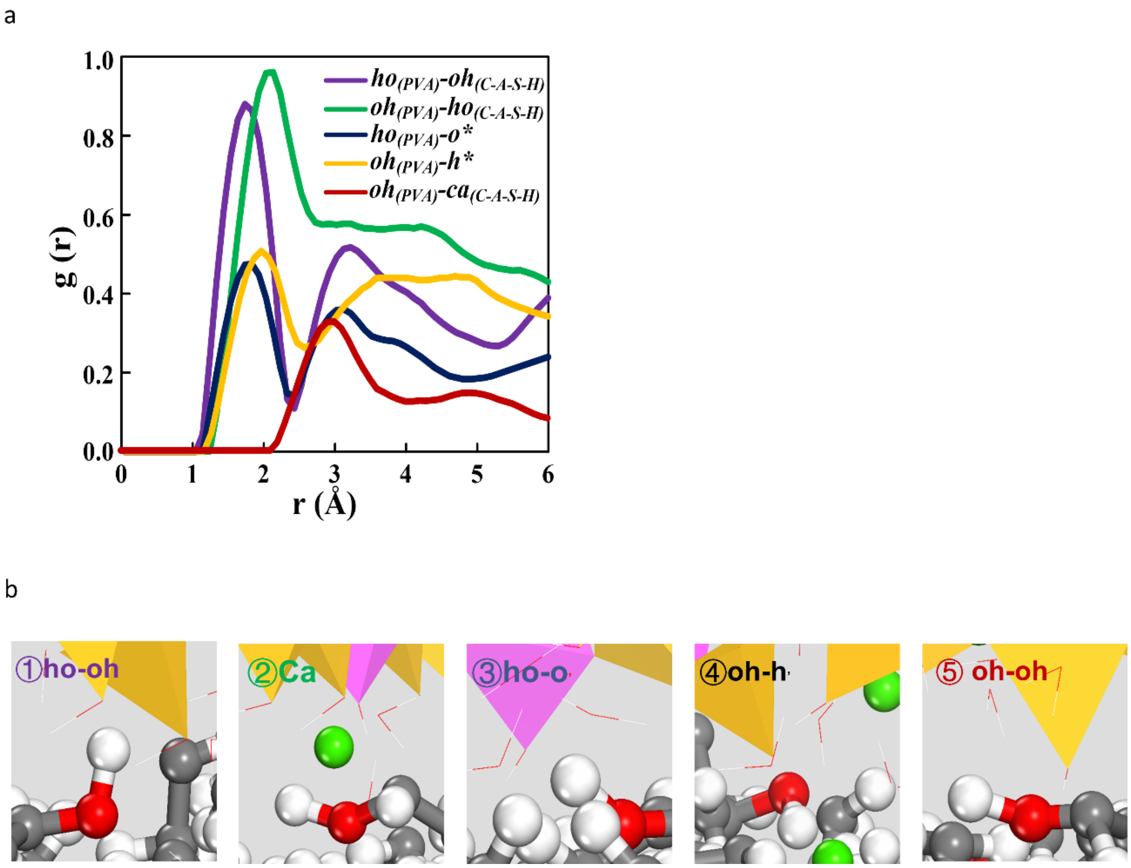

Figure S6. MD simulation results of cement aerogel. a) The radial distribution functions from the model in this simulation: -ho(PVA): hydrogen atoms from PVA chains, -oh(C-S-H):oxygen atoms from C-S-H, -oh(PVA): oxygen atoms from PVA chains, -ho(C-S-H): hydrogen atoms from C-S-H, -o*: oxygen atoms from water, -h*: hydrogen atoms from water, -ca(C-S-H): calcium atoms from C-S-H. b) Snapshots of the connections between PVA and C-S-H, green dotted circle are Ca-O coordination.

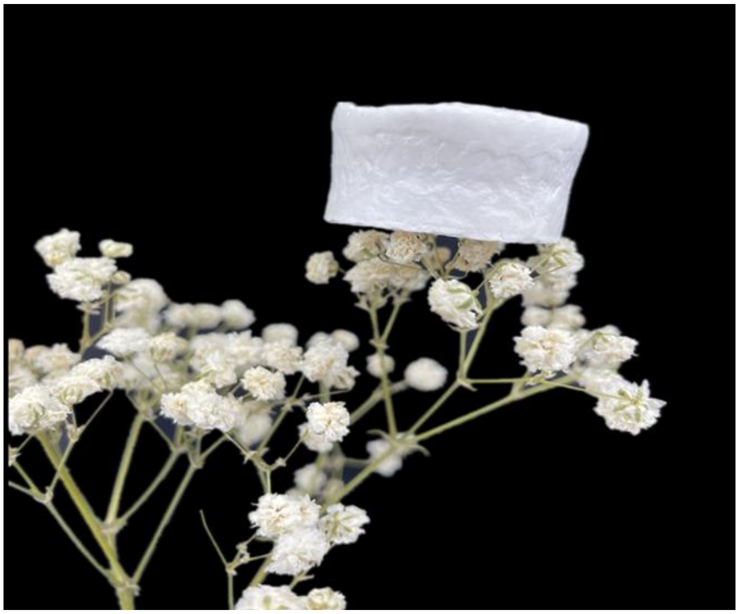

Figure S7. Cement aerogel shows ultralight weight with a density of 0.015 g cm^{-3}.

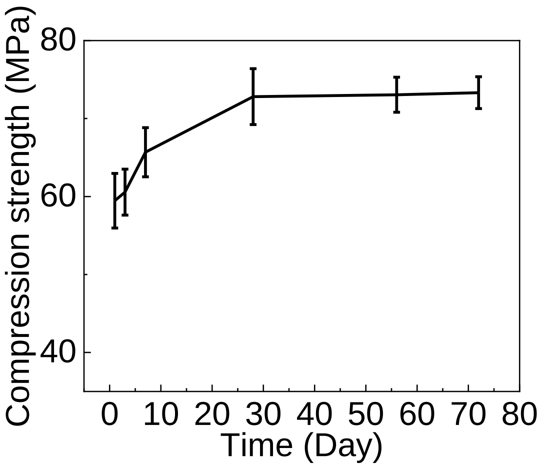

Figure S8. The durability of cement aerogel. Cement aerogel has good long-term durability: compression strength increases from 59.46 MPa to 73.32 MPa.

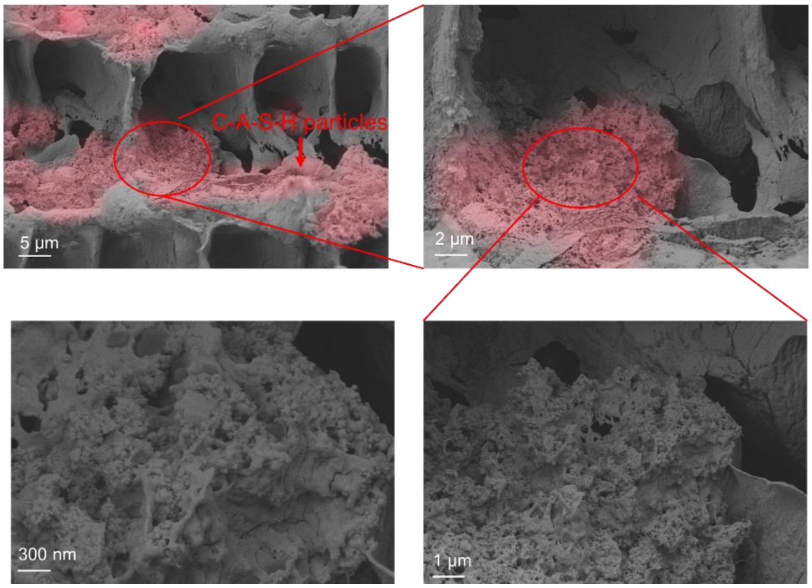

Figure S9. SEM images of cement aerogel interface, showing nanoparticles are tightly bonded on the PVA surface.

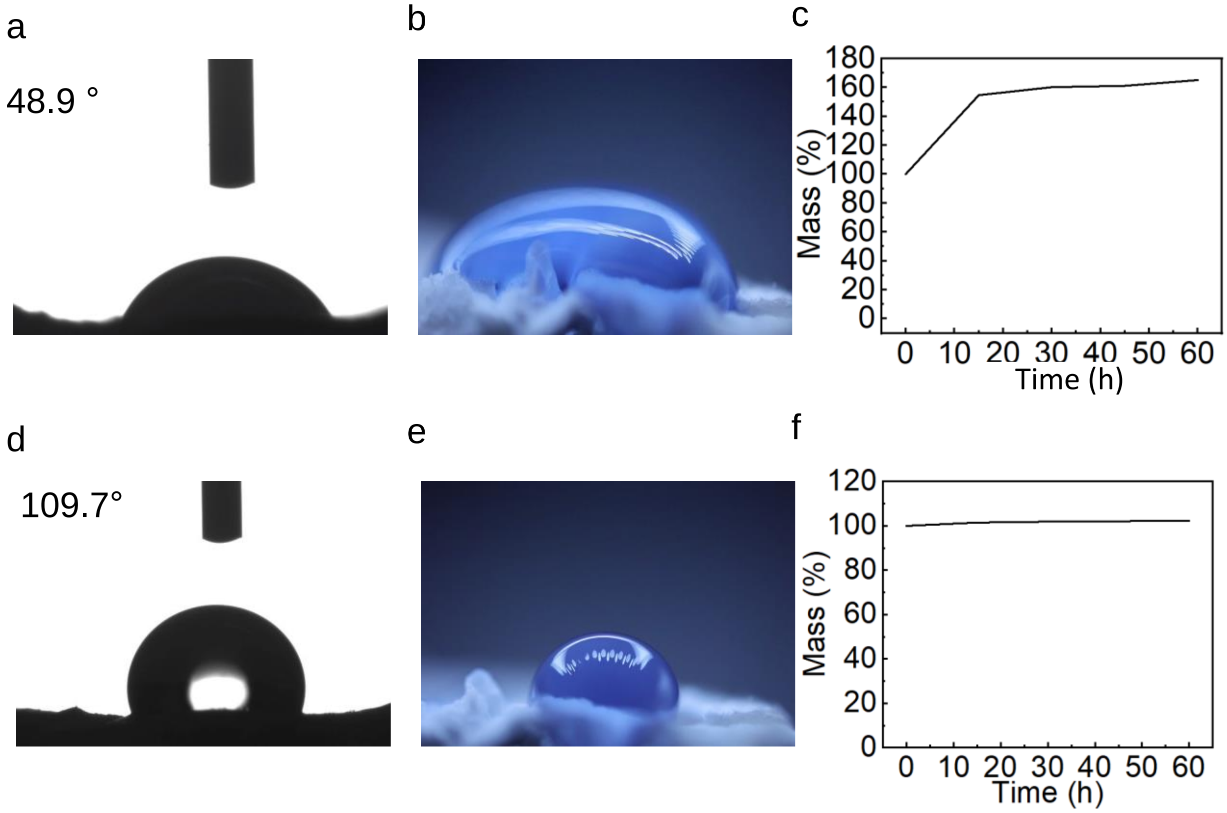

Figure S10. Before coating: a) contact angle. b) micro-optical photo. c) weight change under 100% RH. After coating: d) contact angle. e) micro-optical photo. f) weight change under 100% RH.

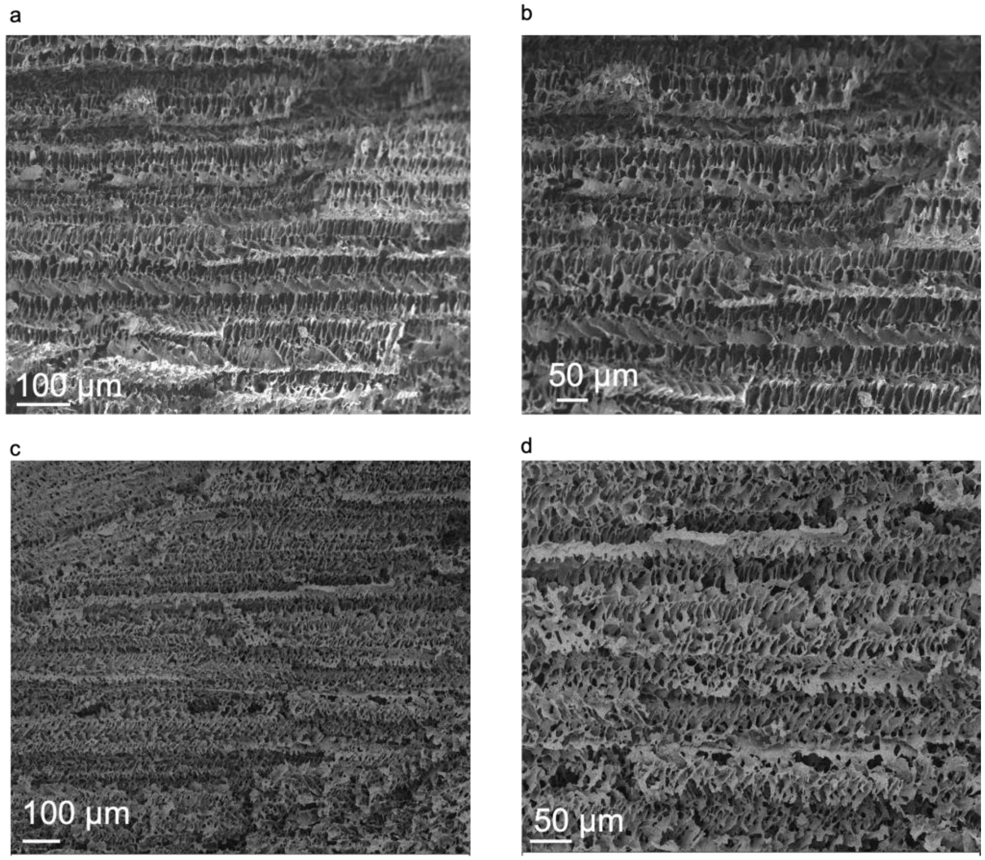

Figure S11. SEM images of cement aerogel a) b) before impact loads, c) d) after impact loads, showing no obvious cracks after impact loads.

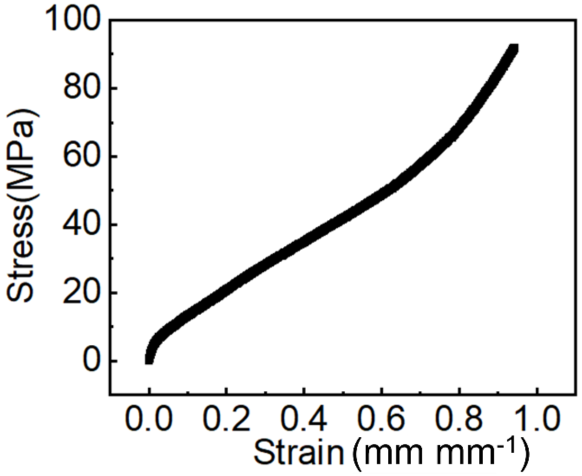

Figure S12. The strain and stress curve of cement aerogel, showing a mechanical stiffness of 294.61 MPa.

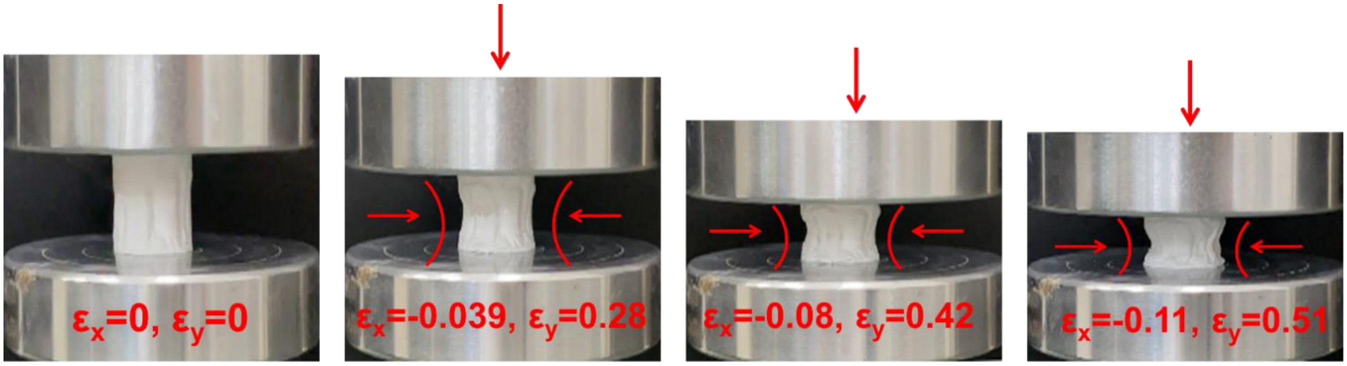

Figure S13. The real-time longitudinal strain evolution of cement aerogel. The sequential pictures show Negative Poisson's ratio (NPR) of cement aerogel under compression test. NPR as high as -0.22 can be clearly observed from compression tests.

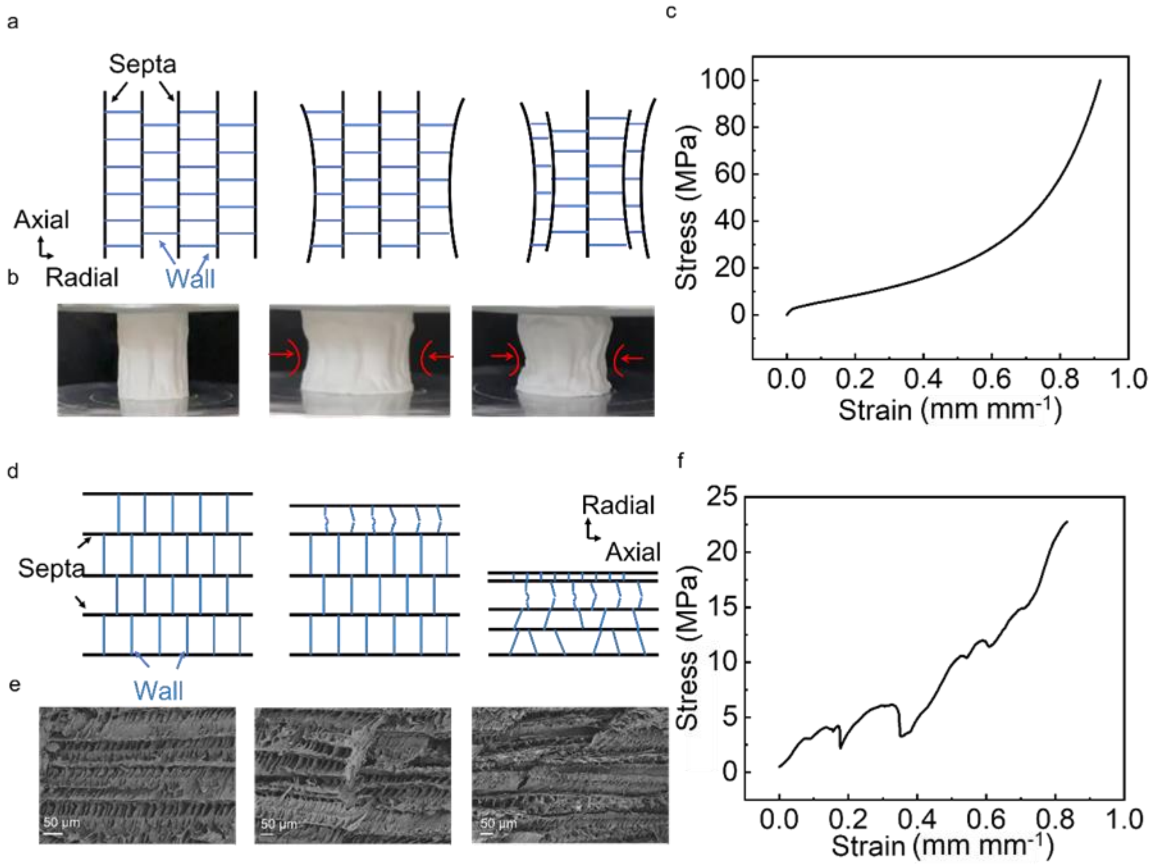

Figure S14. The anisotropic mechanical property of cement aerogel. At the radial direction: a) Schematic of mechanical failure. b) Negative Poisson's ratio behavior. c) Strain and stress curve under compression. At the axial direction: d) Schematic of mechanical failure. e) Sequential SEM images of in situ compressed cement aerogel. f) Strain and stress curve under compression.

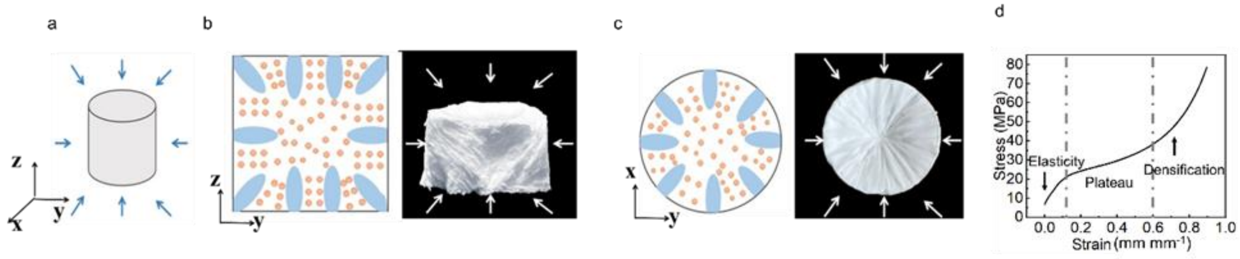

Figure S15. a) Schematic of 3D controlled ice freezing template. b) Schematic of controlled ice crystals in y-z view. c) Radially prepared sample in x-y view. d) Strain-stress curve of cement aerogel under compression: liner elasticity, plateau and high densification. The elastic modulus of this sample is 305.80 MPa. The compressive strength at 80% strain is 58.86 MPa. The toughness of this cement aerogel is 3.5 MJ m^{-3} at 15% strain.

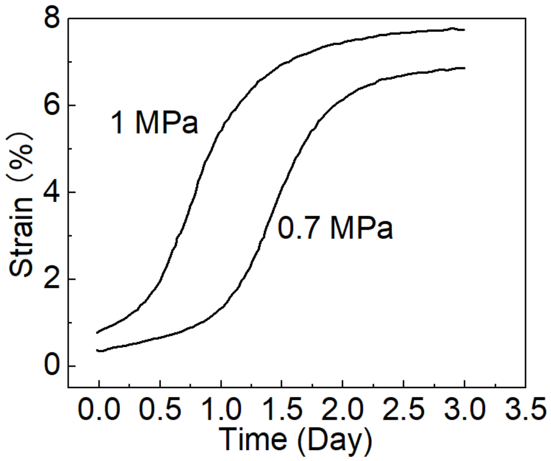

Figure S16. Creeping behavior of cement aerogel, which can relieve the internal stress and effectively prevent cracking.

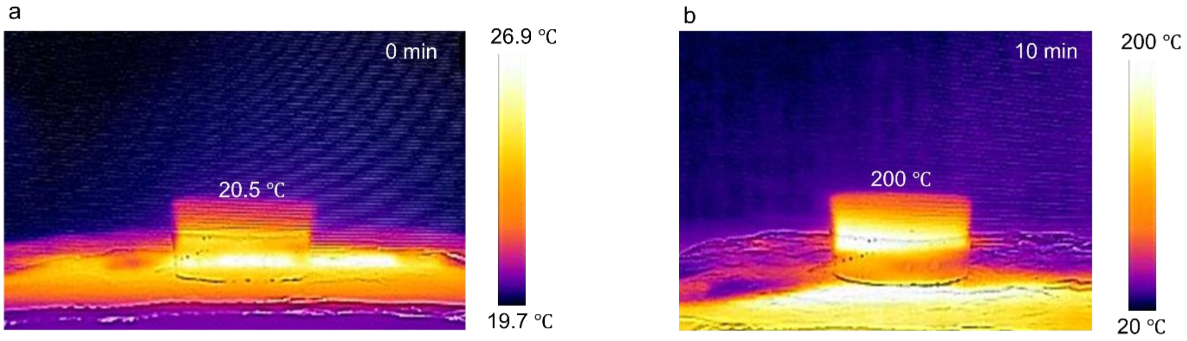

Figure S17. The infrared images of pure cement samples during hot plat experiment. a) 0 min after 200 °C heating. b) 10 min after 200 °C heating.

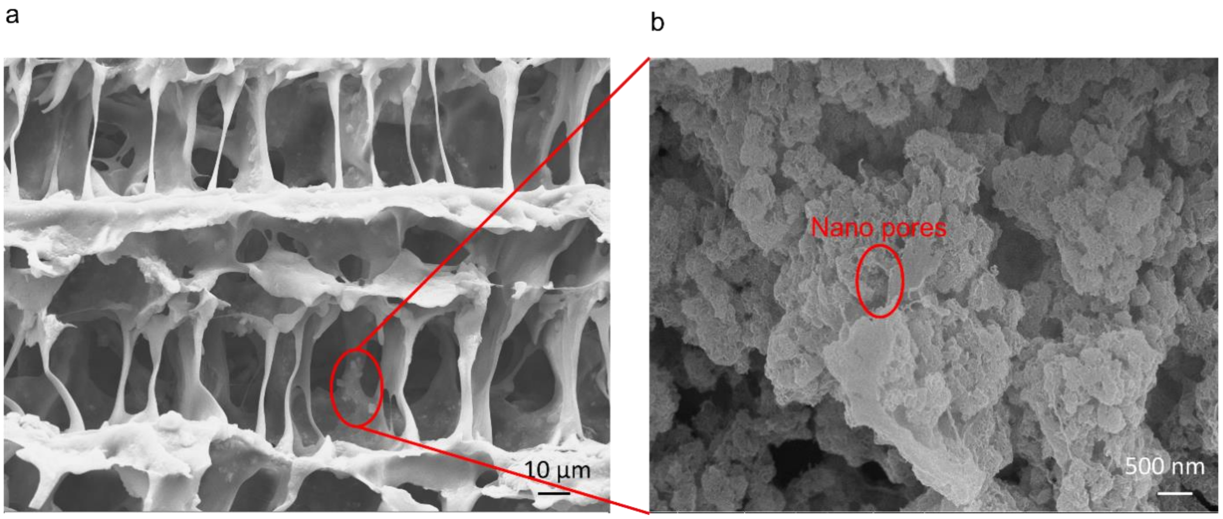

Figure S18. SEM pictures of cement aerogel. a) SEM picture of cement aerogel at micro scale. b) SEM picture of cement aerogel at nano scale. Cement aerogel has two levels pores: the skeleton with layer spacing of 20-50 µm and the C-A-S-H nanoparticles with 10-50 nm space wrapped on the surface of skeleton.

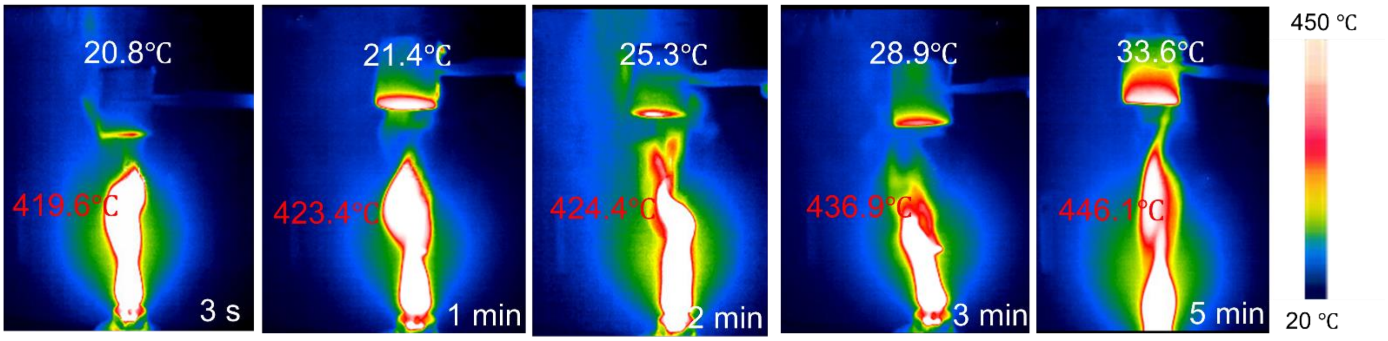

Figure S19. Infrared images of the cement aerogel under fire, showing a low temperature at 33.6 °C after 5-minute fire exposure.

Part 2: Tunability Section

Note S1

To showcase the tunability of the composition of the cement aerogel, different masses of 100 g PVA with concentrations of 4.5 wt%, 7.5 wt%, 10 wt% and 50 g $Ca(NO_3)_2$ with concentrations of 0.3 M, 0.65 M and 1.0 M solution were mixed respectively. Furthermore, 40 g Na_2SiO_3 with concentrations of 0.3 M, 0.65 M, 1.0 M and 10 g $Al(NO_3)_3$ with concentrations of 0.3 M, 0.65 M, 1.0 M were added to the mixtures. The resulting cement aerogels have densities ranging from 0.015 g cm^{-3} to 0.84 g cm^{-3}, corresponding to porosities from 90.43% to 79.83%, details as shown in Table S1 and Table S2. Furthermore, Table S3 presents the results of mechanical strength and thermal conductivity from cement aerogels at different mixing ratios. Note that the decreasing thermal insulation from ratio A to ratio C is likely due to the higher composition of PVA, as PVA is more thermally conductive than C-A-S-H. Therefore, the cement aerogel with ratio A composition is selected for further characterization and demonstration.

Table S1 The composition of cement wood.

No.	PVA	$Ca(NO_3)_2$	Na_2SO_3	$Al(NO_3)_3$
Ratio A	100 g (10 wt%)	50 g (1 M)	40 g (1 M)	10 g (1 M)
Ratio B	100 g (7.5 wt%)	50 g (0.65 M)	40 g(0.65 M)	10 g (0.65 M)
Ratio C	100 g (4.5 wt%)	50 g (0.3 M)	40 g (0.3 M)	10 g (0.3 M)

Table S2 The density and porosity of cement aerogel at different mixing ratios.

No.	Density (mg cm^{-3})	Porosity (%)
Ratio A	84.36±2.25	79.83
Ratio B	35.28±1.35	86.44
Ratio C	15.68±0.21	90.43

Table S3 The performance results of cement aerogel at different mixing ratios.

No.	Thermal conductivity (W m^{-1} K^{-1})	Mechanical strength (MPa) at 80% strain
Ratio A	0.025	59.46
Ratio B	0.027	33.95
Ratio C	0.028	23.36

Part 3: Table

Table S4 The thermal conductivity of different commercial insulations

Materials	Thermal conductivity (W m^{-1} K^{-1}))
Brick	0.60
Natural Rubber	0.58
Epoxy	0.21
Wood (radial)	0.10
Rock wood	0.04
Foam concrete	0.04
Fiber glass	0.04
Polystyrene (Styrofoam)	0.033
This work	0.025

Part 4: Movies

Movie S1. The 3D reconstruction of cement aerogel through XCT.

Movie S2. The compression test of cement aerogel, the clear NPR behavior can be observed.

Movie S3. The impact load test of cement aerogel. When a 200 g steel ball falls from 30 cm height three times, there is no visible crack can be seen in cement aerogel.

Movie S4. The burning test of cement aerogel. After 5 min fire, there is no apparent damage in cement aerogel.
